# Epidemiology and Burden of Bloodstream Infections Caused by Extended-Spectrum Beta-Lactamase Producing *Enterobacteriaceae* in a Pediatric Hospital in Senegal

**DOI:** 10.1371/journal.pone.0143729

**Published:** 2016-02-11

**Authors:** Awa Ndir, Amadou Diop, Pape Makhtar Faye, Moussa Fafa Cissé, Babacar Ndoye, Pascal Astagneau

**Affiliations:** 1 PhD Program, Université Pierre Marie Curie, Paris, France; 2 Institut Pasteur de Dakar, Dakar, Sénégal; 3 Hôpital pour enfants Albert Royer, Dakar, Sénégal; 4 National nosocomial infection program, Ministry of Health, Dakar, Sénégal; 5 Universités Sorbonne Paris-Cité, Paris, France; Université d'Auvergne Clermont 1, FRANCE

## Abstract

**Context:**

Severe bacterial infections are not considered as a leading cause of death in young children in sub-Saharan Africa. The worldwide emergence of extended-spectrum beta-lactamase producing *Enterobacteriaceae* (ESBL-E) could change the paradigm, especially in neonates who are at high risk of developing healthcare-associated infections.

**Objective:**

To evaluate the epidemiology and the burden of ESBL-E bloodstream infections (BSI).

**Methods:**

A case-case-control study was conducted in patients admitted in a pediatric hospital during two consecutive years. Cases were patients with *Enterobacteriaceae* BSI and included ESBL-positive (cases 1) and ESBL-negative BSI (cases 2). Controls were patients with no BSI. Multivariate analysis using a stepwise logistic regression was performed to identify risk factors for ESBL acquisition and for fatal outcomes. A multistate model was used to estimate the excess length of hospital stay (LOS) attributable to ESBL production while accounting for time of infection. Cox proportional hazards models were performed to assess the independent effect of ESBL-positive and negative BSI on LOS.

**Results:**

The incidence rate of ESBL-E BSI was of 1.52 cases/1000 patient-days (95% CI: 1.2–5.6 cases per 1000 patient-days). Multivariate analysis showed that independent risk factors for ESBL-BSI acquisition were related to underlying comorbidities (sickle cell disease OR = 3.1 (95%CI: 2.3–4.9), malnutrition OR = 2.0 (95%CI: 1.7–2.6)) and invasive procedures (mechanical ventilation OR = 3.5 (95%CI: 2.7–5.3)). Neonates were also identified to be at risk for ESBL-E BSI. Inadequate initial antibiotic therapy was more frequent in ESBL-positive BSI than ESBL-negative BSI (94.2% versus 5.7%, p<0.0001). ESBL-positive BSI was associated with higher case-fatality rate than ESBL-negative BSI (54.8% versus 15.4%, p<0.001). Multistate modelling indicated an excess LOS attributable to ESBL production of 4.3 days. The adjusted end-of-LOS hazard ratio for ESBL-positive BSI was 0.07 (95%CI, 0.04–0.12).

**Conclusion:**

Control of ESBL-E spread is an emergency in pediatric populations and could be achieved with simple cost-effective measures such as hand hygiene, proper management of excreta and better stewardship of antibiotic use, especially for empirical therapy.

## Introduction

Lower-middle-income countries account for over 75% of the 10.6 million annual deaths in children under five worldwide [1]. In Africa, the most frequent fatal outcomes stem from neonatal causes (26%), respiratory tract infections (21%), malaria (18%), diarrhea (16%) and HIV-infection (6%) [1]. Bacterial infections are often underreported among causes of child mortality in developing countries, the most frequently reported being upper respiratory tract infections. Bloodstream infections (BSI) are considered as a leading cause of severe bacterial infections in children. These severe sepses are challenging since the disease can progress rapidly to death if prompt effective antibiotic therapy is not undertaken. A few studies in Africa reported high case-fatality rate associated with BSI, mainly caused by Gram-negative bacteria, and being 2-fold higher than that of malaria (43.5% versus 20.2%) [2,3].

BSI are also absent from the World Health Organization estimates of the most frequently reported causes of deaths in neonates which are preterm (28%), sepsis or pneumoniae (26%) and asphyxia (23%) [4]. However, a recent report estimated the incidence of possible severe neonatal bacterial infections of about 6.9 million of annual cases and showed that they were a major contributor to mortality. Indeed they could cause up to one third of the 2.9 million neonatal deaths reported in lower-middle-income countries (LMIC), which account for 99% of the annual worldwide neonatal deaths [4,5].

The worldwide increase of extended-spectrum beta-lactamases produced by *Enterobacteriaceae* (ESBL-E) is worrisome in clinical practice since it may worsen the burden of severe sepsis of young infants in developing countries. Indeed, such highly resistant pathogens are usually isolated from healthcare-associated infections and are associated with poor outcomes [6–13]. The situation is even more alarming with the emergence of new mechanism of resistance reported in ESBL-E strains conferring resistance to carbapenems, which are drugs of last resort to treat ESBL-E infections [14].

Although previous studies already reported the existence of ESBL-E in several African countries, the extent of the ESBL-E epidemic, as well as its determinants and outcomes have been poorly evaluated until now. Based on a clinical study conducted in a large pediatric center in Dakar, Senegal, we aim to determine the epidemiological aspects and estimate the clinical burden of ESBL-E infections in young hospitalized infants.

## Materials and Methods

### Study design and population

Albert Royer Children’s hospital is the referral tertiary-care teaching pediatric hospital in Dakar accounting for 5,000 admissions per year with 120 beds of neonatal, pediatric and surgical activity. A case-case-control study nested in a cohort was conducted including all patients admitted at hospitals during a two-year period (January 1, 2012 to December 31, 2013). Patients from whom an *Enterobacteriaceae* was recovered from a blood sample, drew in case of infection suspicion, were eligible to be included in the cohort study. Only hospital-acquired (HA) BSI caused by *Enterobacteriaceae* strains were considered for data analysis. Indeed, patients with a community-acquired and those with a BSI caused by a bacterial other than an *Enterobacteriaceae* were excluded of the study. The date of BSI onset was the date of collection of the first blood sample yielding an *Enterobacteriaceae* strain. If a bacterial strain was isolated on several occasions, only the first isolation was considered.

Cases were patients with a HA-BSI caused by an *Enterobacteriaceae* and included two subgroups of inpatients: those with ESBL-positive BSI and those with ESBL-negative BSI. Controls were patients who did not experience an infection during the study period and were randomly selected from the hospital database system. Patients who did not have a diagnostic sample drawn during the hospital stay were eligible to be included in the group of control-patients. Among them, patients who did not have a clinical sign of systemic infection and antibiotic prescriptions during the hospital stay were included in the group of control-patients. For each case-patient two control-patients admitted on the same day and in the same unit were selected.

All patients included in the study were followed from admission to discharge or in-hospital death. For each study patient, the following variables were collected: gender, age, germs isolated, resistance profile, underlying comorbidities, diagnosis at admission, interventions related to patient care such as surgical intervention and invasive procedures, length of stay (LOS), date of BSI onset, in-hospital mortality and antibiotics prescribed empirically.

### Definitions

A BSI was suspected in the presence of fever (> = 38°C), hypothermia (<36°C) and other signs of systemic inflammatory response syndrome as detailed in the WHO guideline of the Integrated Management of Childhood Illness [15]. A blood sample was drawn from all inpatients with a suspected BSI. A BSI was defined by the presence of a bacterial strain recovered from blood samples. The infection was considered as hospital acquired if bacterial strain was recovered from blood samples drew at least 48h after the admission of patients and at least 72h after admission of neonates (aged between 1–28 days of life). BSI was considered as community-acquired if bacterial strain was recovered from blood samples drew within the 48h after admission (72h for neonates) and if the patient was not referred from another hospital, otherwise the infection was considered as hospital-acquired.

BSI was defined as ESBL-positive when the blood sample yielded an ESBL-producing *Enterobacteriaceae* and ESBL-negative when the strain isolated was an *Enterobacteriaceae* susceptible to beta-lactams.

Antibiotic therapy was defined as empirical if prescribed initially before susceptibility test results were available. Empirical antibiotic therapy was considered inadequate when the initial antibiotic drug prescribed was not active against the pathogen causing the infection.

### Microbiological methods

*Enterobacteriaceae* strains were identified with API 20E strips (bioMérieux, Marcy l’Etoile, France). Minimum inhibitory concentration was not determined to define drug activity. Susceptibilities against antimicrobial agents and the ESBL production was routinely detected by the double disc diffusion method using antibiotic discs of cefepime, cefotaxime and ceftazidime placed at a distance of 30 mm around a disc of clavulanic acid as recommended by the Antibiogram Committee of the French Microbiology Society [16].

### Statistical analysis

Continuous variables were compared using Wilcoxon rank sum test or Student’s t test if appropriate. In case of non-homogeneity of variances, the 2-tailed test was done by default and the Welch correction was applied. Fisher’s exact test was used for the comparison of categorical variables. To evaluate risk factors to acquire an ESBL-E BSI 2 multivariate models were tested using a backward stepwise logistic regression analysis including all variables with a p-value less than 0.20 in the univariate analysis. Patients with ESBL-positive BSI were compared with patients with ESBL-negative BSI in model 1 then, compared with control-patients (uninfected patients) in model 2. A multivariate analysis using a backward stepwise logistic regression analysis was also performed to assess the risk factors for fatal outcomes. P value less than 0.05 was considered as significant in the whole analysis.

**Fig 1:**
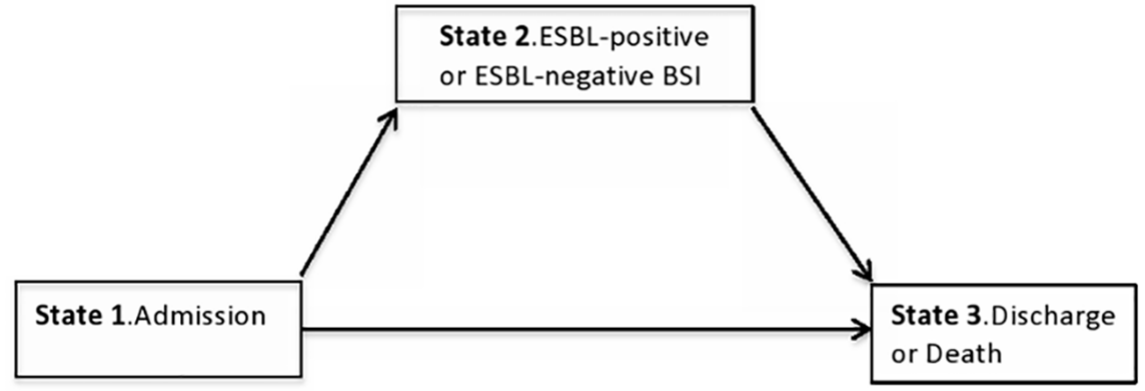
Multistate modelling used for the excess length of stay analysis. Patients enter the model in state 1 at hospital admission, make a transition into state 2 at the time of infection (whether ESBL-positive or ESBL-negative BSI) then move to the absorbing state 3 at the time of discharge or death. Patients who do not experience an infection during their hospital stay move directly from state 1 to state 3. **BSI:** Bloodstream infection. **ESBL-positive**: *Enterobacteriaceae* producing extended spectrum beta-lactamase. **ESBL-negative**: *Enterobacteriaceae* susceptible to beta-lactams.

A multistate model was used to estimate the excess LOS attributable to ESBL production [17,18]. The occurrence of BSI (whether ESBL-positive or ESBL-negative) was the time-dependent exposure, while discharge (alive or dead) was the study endpoint (Fig 1). Non-parametric estimation of transition probabilities between states was performed using the Aalen-Johansen estimator [18–20]. The mean difference in LOS was computed for each day of the interval, as the difference between the estimated LOS given the intermediate state had been reached or not up to that day. The excess LOS attributable to ESBL production was the difference between LOS due to ESBL-positive and LOS due to ESBL-negative BSI. When assessing the LOS due to ESBL-positive BSI, patients with ESBL-positive BSI were compared to patients who did not experience a BSI during hospital stay and patients with ESBL-negative BSI but the latter were administratively censored from the date of their infection. Likewise, patients with ESBL-positive BSI were administratively censored when assessing the LOS due to ESBL-negative BSI. Standard errors and confidence intervals were calculated by 500 bootstrap resampling runs.

To assess the independent effect of ESBL-positive and ESBL-negative BSI on LOS, they were evaluated as time-dependent covariates using multivariate Cox regression analysis to estimate the end-of-LOS hazard ratio (HR). Variables for adjustment included age, newborn, malnutrition, sickle cell disease and mechanical ventilation.

LOS analysis was performed using R, version 2.15.3, an open-source language for statistical computing and graphics. All other analysis were performed using Stata software, release 13.0.

### Ethical considerations

The medical advisory board of Albert Royer Hospital approved this retrospective study. All data collected were anonymized prior to analysis.

## Results

### Characteristics of the study patients

**Fig 2:**
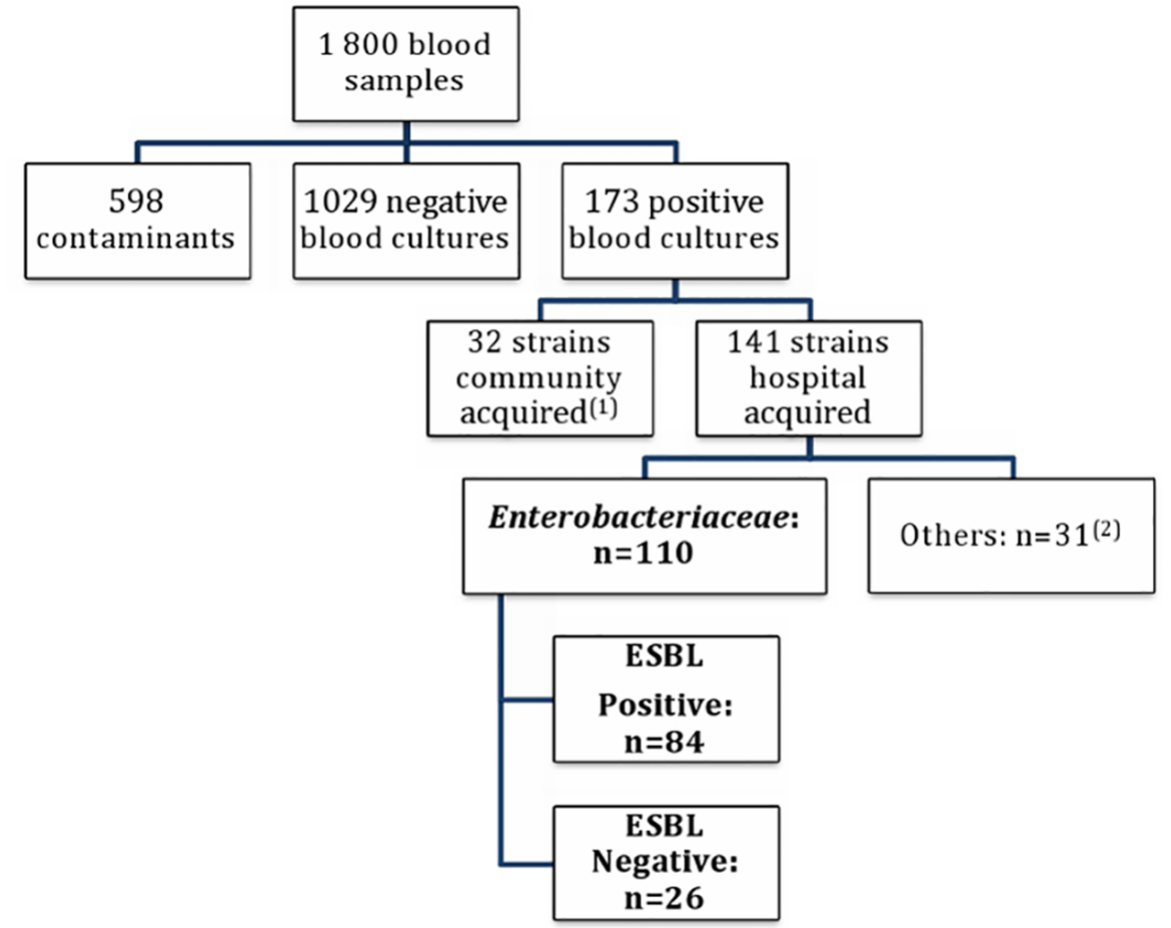
Flow diagram of study patients’ selection. (1): Strains associated with community-acquired BSI: *Enterobacteriaceae* (21), *Pseudomonas aeruginosa (*2), *Staphylococcus spp* (6), *Streptococcus spp (3)*. (2): Strains associated with hospital-acquired BSI: *Pseudomonas aeruginosa* (6), *Staphylococcus aureus* (22), *Streptococcus* spp (3).

During the study period, blood samples were drawn from 1,800 patients with suspected BSI of which 173 (10%) yielded a bacterial strain. Contaminants were found in 36.8% of blood cultures (Fig 2). BSI was hospital-acquired (HA) in 81.5% of cases (n = 141). The epidemiology of bacterial strains involved in BSI cases described in table 1 showed that *Enterobacteriaceae* was mainly recovered from blood cultures. Besides, ESBL-E was the major resistant strain isolated and was more frequent in hospital than community-acquired BSI (59.6% versus 34.4, p = 0.010) (Table 1).

**Table 1:** Characteristics of the study patients with community and hospital-acquired bloodstream infections (n = 173 patients).

Characteristics	TOTAL (n = 173)	Source of the infection		P value
		Hospital-acquired BSI (n = 141)	Community-acquired BSI (n = 32)	
**Demographics**				
Male Sex	91(52.6)	71(50.3)	20(62.5)	0.243
Mean, years (range)	3.3(0–17)	3.3 (0–16)	3.6(0–17)	0.214
**Unit of hospitalization**				
Pediatrics	108(62.4)	89(63.1)	19(59.4)	0.687
Surgery	29(16.8)	22(15.6)	7(21.9)	0.687
Neonatology	36(20.8)	30(21.3)	6(18.7)	0.687
**Pathogens isolated, n (%)**				
Enterobacteriaceae	131(75.7)	110 (78.1)	21(65.6)	0.182
Pseudomonas aeruginosa	8(4.6)	6(4.2)	2(6.2)	0.182
Staphylococcus aureus	28(16.2)	22(15.6)	6(18.7)	0.182
Streptococcus	6(3.5)	3(2.1)	3(9.4)	0.182
**Resistant strains**				
ESBL-E	95(54.9)	84(59.6)	11(34.4)	0.010
MRSA	2(1.1)	2(1.4)	0(0)	

**ESBL-E:** Extended-spectrum beta-lactamase producing *Enterobacteriaceae*

**MRSA**: Methicillin-resistant *Staphylococcus aureus*

The overall incidence rate of HA-BSI caused by ESBL-E strains was of 1.52 cases / 1000 patient-days (95% CI: 1.2–5.6 cases per 1000 patient-days). ESBLs were produced by 88% of *Enterobacter spp* isolates, 82% of *Klebsiella spp* isolates and 58.3% of *E*.*coli* isolates (Table 2).

**Table 2:** Proportion of ESBL producing *Enterobacteriaceae* among hospital-acquired BSI (n = 110 patients).

Pathogens	Number of isolates n (%)	ESBL-E ^a^ n (%)
*Enterobacter spp*	50(45.5)	44(88.0)
*Klebsiella spp*	40(36.4)	33(82.5)
*Escherichia coli*	12(10.9)	7(58.3)
*Salmonella spp*	4(3.6)	0
*Citrobacter freundii*	2(1.8)	0
*Proteus mirabilis*	2(1.8)	0
**Total**	**110 (100.0)**	**84(76.4)**

^a^ Proportion of ESBL strains per species (percentages were calculated by row)

Characteristics of case-patients (n = 110) and controls (n = 220) are detailed in table 3. Cases were constituted of 84 patients with ESBL-positive BSI and 26 patients with ESBL-negative BSI (Table 3).

**Table 3:** Factors associated with ESBL-E BSI: results of univariate analyses comparing ESBL-positive BSI with ESBL-negative BSI (model 1) and ESBL-positive BSI with control-patients (model 2).

Variable, n (%)	ESBL+ (n = 84)	ESBL–(n = 26)	Controls (n = 220)	P-value 1^a^	P-value 2^b^
**Demographics**					
Male	44(52.4)	14(53.8)	111(50.4)	1	0.798
Mean age, years	2.5	4.4	3.7	0.021	0.016
Newborn	27(32.1)	2(7.7)	48(21.9)	0.012	0.074
Prematurity	7(8.3)	2(7.7)	4(1.8)	1	0.012
**Comorbidity**					
Sickle cell disease	28(33.3)	3(11.5)	7(3.2)	0.044	<0.0001
Malnutrition	32(38.1)	4(15.4)	9(4.1)	0.034	<0.0001
Severe anemia	16(19.0)	8(30.8)	31(14.1)	0.276	0.291
**Diagnosis at admission**					
Severe malaria	11(13.1)	4(15.4)	34(15.4)	0.750	0.719
Gastroenteritis	4(4.8)	8(30.8)	65(29.5)	0.0009	<0.0001
Respiratory disease	25(29.8)	8(30.8)	40(18.2)	1	0.041
Cardiovascular disease	3(3.5)	1(3.8)	4(1.8)	1	0.399
Meningitis	5(5.9)	1(3.8)	7(3.2)	1	0.323
Ear, nose and throat disease	10(11.9)	3(11.5)	24(10.9)	1	0.839
**Invasive procedure**					
Surgical intervention	15(17.8)	5(19.2)	36(16.4)	1	0.734
Parenteral nutrition	24(28.6)	10(38.5)	22(10.0)	0.343	<0.0001
Mechanical ventilation	53(63.1)	14(38.5)	32(14.5)	0.040	<0.0001
Catheter	45(53.6)	11(42.3.)	16(7.3)	0.373	<0.0001
Blood transfusion	16(19.0)	3(11.5)	12(5.5)	0.554	0.0006

^a^ P-value 1 refer to model 1 in which ESBL-positive BSI are compared with ESBL-negative BSI

^b^ P-value 2 refer to model 2 in which ESBL-positive BSI are compared with control-patients (uninfected patients)

### Risk factors for ESBL-E BSI

Univariate analysis presented in table 3 showed that patients with ESBL-positive BSI were significantly younger than patients with ESBL-negative BSI (2.5 years versus 4.4 years, p = 0.021). Besides, they were more likely to suffer from sickle cell disease (33.3% versus 11.5%, p = 0.044), to be malnourished (38.1% versus 15.4%, p = 0.034) and to be under mechanical ventilation (63.1% versus 38.5%, p = 0.040) than ESBL-negative BSI. Same differences were observed when comparing patients with ESBL-positive BSI with control-patients (Table 3). Additional factors related to healthcares, such as parenteral nutrition and the use of catheter, were significantly more frequent in patients with ESBL-positive BSI than in control-patients (Table 3). Multivariate analyses performed in model 1 and model 2 indicated that independent risk factors for ESBL-E BSI acquisition were: to be a newborn (OR = 11.4, 95%CI: 5.7–198, p = 0.041), having a sickle cell disease (OR = 3.1, 95%CI: 2.3–4.9, p<0.0001), being malnourished (OR = 2.0, 95%CI: 1.7–2.6, p<0.0001), and being under mechanical ventilation (OR = 3.5, 95%CI: 2.7–5.3, P<0.0001) (Table 4).

**Table 4:** Risk factors for ESBL-E BSI acquisition: results of multivariate analyses.

Variable including in models	Initial Model 1^a^	Initial Model 2^b^	Final Model 1^a^			Final Model 2^b^		
	P-value	P-value	OR	95% CI	P-value	OR	95% CI	P-value
Newborn	0.012	0.074	2.8	1.9–60	<0.0001	11.4	5.7–198	0.041
Prematurity	-	0.012	-	-	-	4.7	2.5–41.8	0.027
Sickle cell disease	0.044	<0.0001	3.8	2.3–10.3	0.002	3.1	2.3–4.9	<0.0001
Malnutrition	0.034	<0.0001	2.8	1.9–5.3	<0.0001	2.0	1.7–2.6	<0.0001
Parenteral nutrition	-	<0.0001	-	-	-	9.6	4.9–98	0.039
Mechanical ventilation	0.025	<0.0001	6.1	3.1–228.9	0.044	3.5	2.7–5.3	<0.0001
Catheter	0.170	<0.0001				4.0	2.9–6.7	<0.0001
Blood transfusion	0.149	0.0006						

Multivariate analyses were performed using a backward stepwise logistic regression including variables with a p <0.20 in the univariate analyses (Table 3). P-value <0.05 was considered as statistically significant.

^a^**Model 1**: ESBL-positive BSI versus ESBL-negative BSI

^b^**Model 2**: ESBL-positive BSI versus control-patients (uninfected patients)

### Antimicrobial prescriptions

All patients with BSI received an empirical antimicrobial therapy, which was mainly a third generation cephalosporin (90%). Initial antibiotic therapy was inadequate to treat 79.1% of BSI infections (n = 87). Inadequate initial antibiotic therapy (IIAT) was more frequent in ESBL-positive BSI than in ESBL-negative BSI (94.2% versus 5.7%, p<0.0001). Besides, patients who received an IIAT were more likely to have a fatal outcome (92% versus 8%, p = 0.0019) and a longer hospital stay (22.9 days versus 14.2 days, p = 0.013) than patients who received an adequate empirical therapy.

### Fatal outcomes

Fifty patients with a BSI caused by an *Enterobacteriaceae* died during the study period (45.4%). The case-fatality rate was significantly higher in ESBL-positive BSI (54.8%) than in ESBL-negative BSI (15.4%) (p<0.001). Univariate analysis showed that fatal outcomes more frequently occurred in young children, in newborns and patients being under mechanical ventilation (Table 5). Additionally, patients who died were more likely to have an ESBL-E BSI and to receive an inadequate initial antibiotic therapy than patients who survived. In the multivariate analysis, ESBL production remained the significant independent risk factor for death (OR = 2.9, 95% CI: 1.8–7.3, p = 0.001).

**Table 5:** Factors associated with fatal outcomes: results of univariate and multivariate analysis.

	Fatal outcome		Univariate analysis			Multivariate analysis*		
Variable, n (%)	Yes (n = 50)	No (n = 60)	OR	95% CI	P value	OR	95% CI	P value
**Demographics**								
Male	24(48)	28(46.7)	1.1	0.5–2.2	1			
Mean age, years	1.5	4.2			0.0003			
Newborn	19(38)	10(16.7)	3.1	1.3–7.3	0.016			
Prematurity	6(12)	3(5)	2.6	0.7–9.9	0.295			
**Comorbidity**								
Sickle cell disease	13(26)	18(30)	0.8	0.3–1.9	0.676			
Malnutrition	18(36)	18(30)	1.3	0.6–2.9	0.545			
Severe anemia	10(20)	14(23.3)	0.8	0.3–2.0	0.817			
**Diagnosis at admission**								
Severe malaria	7(14)	8(13.3)	1.1	0.4–3.1	1			
Gastroenteritis	1(2)	11(18.3)	0.1	0–0.6	0.006			
Respiratory disease	19(38)	14(23.3)	2.0	0.9–4.6	0.101	3.0	1.4–17.6	0.093
Cardiovascular disease	2(4)	2(3.3)	1.2	0.2–7.1	1			
Meningitis	5(10)	1(1.7)	6.5	0.9–1.2	0.090			
Ear, nose and throat disease	6(12)	7(11.7)	1.0	0.3–3.1	1			
**Invasive procedure**								
Surgical intervention	7(14)	13(21.7)	0.6	0.2–1.6	0.332			
Parenteral nutrition	12(24)	22(36.7)	0.5	0.2–1.2	0.214			
Mechanical ventilation	35(70)	28(46.7)	2.7	1.2–5.8	0.019	6.5	2.9–34.4	0.098
Catheter	27(54)	29(48.3)	1.2	0.5–2.6	0.572			
Blood transfusion	9(18)	10(16.7)	1.1	0.4–2.9	1			
**ESBL Production**	46(92)	38(63.3)	6.6	2.2–20.0	0.0006	2.9	1.8–7.3	0.001
**Inadequate initial antibiotic therapy**	46(92)	41(68.3)	5.3	1.7–16.1	0.002			

*Multivariate analyses was performed using a backward stepwise logistic regression including variables with a p <0.20 in the univariate analyses. P-value <0.05 was considered as statistically significant.

### Length of stay (LOS)

The mean LOS for patients with ESBL-positive BSI and ESBL-negative BSI was 22.5 days (95%CI: 18-5-26. days) and 12.6 days (95%CI: 9.5–15.8 days) respectively (p<0.0001). The results of the multistate model showed an excess LOS attributable to ESBL production of 4.3 days (Table 6). Besides, ESBL-positive BSI significantly reduced hazard of discharge (dead or alive) after adjustment for confounding (HR = 0.07, [95%CI, 0.04–0.12]) and consequently prolonged LOS.

**Table 6:** Estimation of the excess length of stay (LOS) and hazard ratios (HR) of end-of-LOS associated with ESBL-positive and ESBL-negative BSI.

		End-of-LOS HR	
	Excess LOS (95%CI), days)	Univariate (95% CI)	Multivariate (95% CI)
ESBL-positive BSI ^a^	5.1 (3.82–5.62)	0.18 (0.13–0.24)	0.07 (0.04–0.12)
ESBL-negative BSI ^b^	0.8 (0.74–1)	0.66 (0.44–0.99)	0.12 (0.07–0.21)

**CI**: confidence interval

^a^**Model A**: Excess LOS due to ESBL-positive BSI

Cases: ESBL-positive BSI (n = 84)

Controls: ESBL-negative BSI (n = 26) censored at time of infection + control-patients (uninfected patients) (n = 220)

^**b**^**Model B**: Excess LOS due to ESBL-negative BSI

Cases: ESBL-negative BSI (n = 26)

Controls: ESBL-positive BSI (n = 84) censored at time of infection + control-patients (uninfected patients) (n = 220)

## Discussion

Our study highlights an alarming rate of ESBL production among *Enterobacteriaceae* strains associated with BSI. In addition, a significant part of ESBL-E BSI was severe sepsis associated with fatal outcome and prolonged hospital stay. We have estimated an incidence rate of ESBL-E BSI of 1.52 cases / 1000 patient-days which is higher than those reported through the national surveillance of multidrug-resistant bacteria in France where ESBL-E rate is increasing dramatically since 2003 (0.054/1000 patients-days in 2012) [21,22]. Besides, this ESBL-E incidence is higher than that previously reported in one African healthcare setting [2]. However, the paucity of studies on childhood ESBL-E BSI from Africa does not allow a thorough comparison. To our knowledge, our study is the only one on this topic in sub-Saharan Africa since a Tanzanian study published ten years ago that had much narrower scope [23].

The high rate of ESBL-E strains we have found should be interpreted with caution. Indeed, when an infection is first suspected in primary care settings antibiotic drugs are frequently prescribed without drawing any biological samples since microbiology laboratories are not available in these settings. Thus, patients admitted at hospital if no recovery is observed may receive antibiotic drugs, especially 3^rd^ generation cephalosporins, prior to the hospital admission. In contrast, when an infection was suspected in the hospital study, blood samples were drawn systematically before the initiation of the antibiotherapy. Antibiotic prescriptions prior to blood culture, also reported in another African country [2], may be a selection factor of resistant strains and may limit the detection of susceptible strains. Therefore, the overuse of antibiotic drugs prior to hospital admission may explain the low rate of positive blood cultures we found (10%) and may bias the proportion of ESBL strains. Antimicrobial therapy before hospital admission has already been reported as a major risk factor for ESBL-E acquisition [24–27]. Unfortunately, prescriptions prior to admission were not available in the patients’ medical files and were not collected in our study.

The high incidence rate of ESBL-E BSI also raises the issue of the choice of 3^rd^ generation cephalosporins as systematic empirical treatment. Indeed, empirical antimicrobial therapy was found inadequate to treat the majority of BSI and was more frequent in ESBL-positive BSI than in ESBL-negative BSI. Reports about inadequate initial antibiotherapy in children in Africa are scarce. However, consistent with other studies, we demonstrated that inadequate initial antibiotic therapy (IIAT) was associated with increased case-fatality rate and prolonged hospital stay [28–34]. Therefore, knowledge of the local bacterial epidemiology and their susceptibility patterns is crucial for clinicians and should guide empirical antibiotic therapy prescription. Beta-lactams could therefore be used after determination of antibiotic drug activity. Furthermore, since genes encoding ESBL and other resistance to commonly used antibiotics such as fluoroquinolones are often on the same mobile DNA element, the multidrug-resistant phenotype of ESBL-producing bacteria limits effective therapeutic options and causes a delay in initiating adequate antimicrobial therapy [28,29,32]. In resource-poor countries, some factors such as the lack of functional microbiology laboratory for pathogens detection may hamper the prompt initiation of adequate therapy crucial to treat BSI. When microbiology laboratory exists, mostly in referral tertiary care hospitals, bacteriological results are not available quickly enough to adjust empirical therapy. Additionally, expensive effective therapies to treat ESBL-E infections such as carbapenems are mostly unavailable or beyond the financial reach of most patients living in Senegal.

To our knowledge, risk factors for ESBL-BSI acquisition in children have not been assessed previously in lower-middle-income countries. Using a case-case-control study, we identified several risk factors specifically related to ESBL-E BSI including some related to the underlying disease and others to healthcare procedures. The case-case-control study design is considered to be the most appropriate method when assessing risk factors specific of antibiotic-resistant pathogen acquisition without introducing a potential selection bias [35–38]. Patients who suffered from sickle cell disease or malnutrition were found at risk to acquire an ESBL-E BSI. We also found that being under mechanical ventilation was a risk factor for ESBL-E BSI suggesting a cross-transmission of pathogens. Newborns were also identified as patients at risk to acquire an ESBL-E BSI. This may be explained by their immature immune systems and the overly intensive cares they received, especially for pre-term or low birthweight neonates. ESBL strains may be transmitted from mothers to newborns during delivery; however further studies are required to determine the association between the mother’s colonization and the acquisition of an ESBL-infection by neonates. Poor conditions of clinical cares could also increase this risk. Thus, particular attention should be given to newborns during and after delivery, and during nursing cares. To prevent the transmission of ESBL isolates, training of healthcare workers on standard precautions, such as hand hygiene, should be reinforced as recommended [39–41]. Infection control programs particularly focused on hand hygiene was showed effective to decrease the rate of Methicillin-resistant *Staphylococcus aureus* strains [39]. However additional control measures specifically targeting ESBL strains should be implemented. These measures should focus on the management of excreta since the main reservoir of *Enterobacteriaceae* strains is the human digestive tract.

We also showed a prolonged LOS of 4.3 days attributable to ESBL production. To estimate this excess LOS we used a multistate model, a statistical approach which treats the occurrence of BSI as time-dependent and takes into account competing events thereby avoiding the time-dependent bias inherent in other commonly used statistical methods [17,20,42–45]. Indeed, if the occurrence of the BSI is not explicitly modeled as time-dependent, its impact on length of stay and consequently on hospital costs will inevitably be overestimated [43,44,46]. Our study confirms that multistate modelling is a suitable approach since we found an excess LOS attributable to ESBL production of 4.3 days while taking into account time of infection compared to an excess LOS of 9.9 days using standard technics.

The economic impact of ESBL-E BSI was not the purpose of the present study, however it has been demonstrated elsewhere that the excess length of stay is one main driver of hospital costs and that hospital bed-day cost could represent almost 60% of their total cost [47,48]. Therefore, using the average bed-day hospital cost we estimated from the patient perspective an extra-cost of 75 euros due to the excess LOS attributable to ESBL-E production. This additional cost is substantial given Senegal’s LMIC status where it is close to the mean monthly salary of 87 euros [49]. This estimated financial burden of ESBL-E BSI would be substantially higher if antibiotic therapy were included, especially if this therapy consisted of the costly carbapenems, which remain the only antibiotic therapy effective to treat ESBL-E infections.

## References

[1] Bryce J, Boschi-Pinto C, Shibuya K, Black RE and the WHO Child Health Epidemiology Reference Group. WHO estimates of the causes of death in children. Lancet. 2005; 365:1147–1152. 15794969 10.1016/S0140-6736(05)71877-8

[2] Bloomberg B, Manji KP, Urassa WK, Tamin BS, Mwakagile DM, Jureen R et al Antimicrobial resistance predicts death in Tanzanian children with bloodstream infections: a prospective cohort study. BMC Infect Dis 2007; 7: 43 17519011 10.1186/1471-2334-7-43 PMC1891109

[3] Aiken AM, Mturi N, Njuguna P, Mohammed S, Berkeley JA, Mwangi I et al Risk and causes of paediatric hospital-acquired bacteraemia in Kilifi District Hospital, Kenya: a prospective cohort study. Lancet. 2011; 378: 2021–27. 10.1016/S0140-6736(11)61622-X 22133536 PMC3242162

[4] Lawn JE, Cousens S, Zupan J. Four million neonatal deaths: When? Where? Why? Lancet. 2005; 365:891–900. 15752534 10.1016/S0140-6736(05)71048-5

[5] Seale AC, Blencowe H, Manu AA, Nair H, Bahl R, Qazi SA et al Estimates of possible severe bacterial infection in neonates in sub-Saharan Africa, south Asia, and Latin America for 2012: a systematic review and meta-analysis. The Lancet Infectious Diseases. 2014; 14: 731–741 10.1016/S1473-3099(14)70804-7 24974250 PMC4123782

[6] Rodríguez-Baño J, Pascual A. Clinical significance of extended-spectrum beta-lactamases. Expert Rev Anti Infect Ther. 2008; 6: 671–683. 10.1586/14787210.6.5.671 18847405

[7] Giske CG, Monnet DL, Cars O, Carmeli Y; ReAct-Action on Antibiotic Resistance. Clinical and economic impact of common multidrug-resistant Gram-negative bacilli. Antimicrob Agents Chemother.2008;52:813–821. 18070961 10.1128/AAC.01169-07 PMC2258516

[8] Tumbarello M, Spanu T, Di Bidino R, Marchetti M, Ruggeri M, Trecarichi EM et al. Costs of bloodstream infections caused by Escherichia coli and influence of extended-spectrum beta-lactamase production and inadequate initial antibiotic therapy. Antimicrob Agents Chemother. 2010; 54: 4085–4091. 10.1128/AAC.00143-10 20660675 PMC2944559

[9] Anderson DJ, Engemann JJ., Harrell LJ, Carmeli Y, Reller LB and Kaye KS. Predictors of mortality in patients with bloodstream infection due to ceftazidime-resistant Klebsiella pneumoniae. Antimicrob Agents Chemother. 2006; 5:1715–1720. 10.1128/AAC.50.5.1715-1720.2006 PMC1472233 16641440

[10] Kim BN, Woo JH, Kim MN, Ryu J, and Kim YS. Clinical implications of extended-spectrum beta-lactamase producing Klebsiella pneumoniae bacteremia. Journal Hosp Infect. 2002; 52:99–106. 12392901 10.1053/jhin.2002.1288

[11] Kim YK, Pai H, Lee HJ, Park SE, Choi EH, Kim J, Kim JH, and Kim EC. Bloodstream infections by extended-spectrum β-lactamase-producing Escherichia coli and Klebsiella pneumoniae in children: epidemiology and clinical outcome. Antimicrob. Agents Chemother. 2002; 46:1481–1491. 11959586 10.1128/AAC.46.5.1481-1491.2002 PMC127143

[12] Lautenbach E, Patel JB, Bilker WB, Edelstein PH and Fishman NO. Extended-spectrum β-lactamase-producing Escherichia coli and Klebsiella pneumoniae: risk factors for infection and impact of resistance on outcomes. Clin Infect Dis. 2001; 32:1162–1171. 11283805 10.1086/319757

[13] Schwaber MJ, Navon-Venezia S, Kaye KS, Ben-Ami R, Schwartz D and Carmeli Y. Clinical and economic impact of bacteremia with extended-spectrum beta-lactamase producing Enterobacteriaceae. Antimicrob Agents Chemother. 2006; 50:1257–1262. 16569837 10.1128/AAC.50.4.1257-1262.2006 PMC1426954

[14] Nordmann P, Naas T and Poirel L. Global spread of carbapenemase-producing Enterobacteriaceae. Emerg Infect Dis. 2011; 10:1791–1798. 10.3201/eid1710.110655 PMC3310682 22000347

[15] Young Infants Clinical Signs Study Group. Clinical signs that predict severe illness in children under age 2 months: a multicenter study. Lancet 2008; 371: 135–42 10.1016/S0140-6736(08)60106-3 18191685

[16] Société Française de Microbiologie. European Society of Clinicial Microbiology and Infectious Diseases. Comité de l’antibiothérapie de la Société Française de Microbiologie. Recommendations. 2015. Available from: http://www.sfm-microbiologie.org/UserFiles/files/casfm/CASFM_EUCAST_V1_2015.pdf.

[17] Stewardson A, Fankhauser C, De Angelis G, Rohner P, Safran E, Schrenzel J et al Burden of bloodstream infection caused by extended-spectrum beta-lactamase-producing Enterobacteriaceae determined using multistate modeling at a Swiss University Hospital and a nationwide predictive model. Infect Control Hosp Epidemiol. 2013; 34:133–43. 10.1086/669086 23295559

[18] Green N, Johnson AP, Henderson KL, Muller-Pebody B, Thelwall S, Robotham JV et al Quantifying the burden of hospital-acquired bloodstream infection in children in England by estimating excess length of hospital stay and mortality using a multistate analysis of linked routinely collected data. J Ped Infect Disease.2014:1–8. 10.1093/jpids/piu073 26582869

[19] Aalen O, Johansen S. An empirical transition matrix for non-homogeneous Markov chains based on censored observations. Scand J Stat 1978;5:141–150.

[20] Beyersmann J, Wolkewitz M, Allignol A, Grambauer N, Schumacher M. Application of multistate models in hospital epidemiology: advances and challenges. Biom J. 2011;53:332–350 10.1002/bimj.201000146 21374697

[21] Carbonne A, Arnaud I, Maugat S, Marty N, Dumartin C, Bertrand X et al on behalf of the MDRB Surveillance National Steering Group (BMR-Raisin). National multidrug-resistant bacteria (MDRB) surveillance in France through the RAISIN network: a 9 year experience. J Antimicrob Chemother.2013; 68:954–959. 10.1093/jac/dks464 23194721

[22] Réseau d’alerte d’Investigation et de Surveillance des Infections Nosocomiales (RAISIN). Surveillance des bactéries multirésistantes dans les établissements de santé en France. Résultats 2011 Available from: http://www.cclinparisnord.org/BMR/BMR2011.pdf

[23] Blomberg B, Jureen R, Manji KP, Tamim BS, Mwakagile DS, Urassa WK et al High rate of fatal cases of pediatric septicemia caused by gram-negative bacteria with extended-spectrum beta-lactamases in Dar es Salaam, Tanzania. J Clin Micro. 2005, 43:745–749. 10.1128/JCM.43.2.745-749.2005 PMC548071 15695674

[24] Kaya O, Akcam FZ, Gonen I, Unal O and Ceylan T. Risk factors for bacteremia due to extended-spectrum beta-lactamase-producing Escherichia coli in a Turkish hospital. J Inf Dev Countries. 2013;7:507–512. 10.3855/jidc.2788 23857384

[25] Pena C, Pujol M, Ricart A, Ardanuy C, Ayats J, Linares J et al Risk factors for faecal carriage of Klebsiella pneumoniae producing extended spectrum beta-lactamase (ESBL-KP) in the intensive care unit. J Hosp Infect. 1997; 35:9–16. 9032631 10.1016/s0195-6701(97)90163-8

[26] Tumbarello M, Spanu T, Sanguinetti M, Citton R, Montuori E, Leone F et al Bloodstream infections caused by extended-spectrum beta-lactamase-producing Klebsiella pneumoniae: risk factors, molecular epidemiology, and clinical outcome. Antimicrob Agents Chemother. 2006; 50:498–504. 16436702 10.1128/AAC.50.2.498-504.2006 PMC1366869

[27] Tsai MH, Chu SM, Hsu JF, Lien R, Huang HR, Chiang MC et al Risk Factors and outcomes for multidrug-resistant gram-negative bacteremia in the NICU. Pediatrics.2014;133:e322–e329. 10.1542/peds.2013-1248 24420803

[28] Hyle EP, Lipworth AD, Zaoutis TE, Nachamkin I, Fishman NO, Bilker WB et al Risk factors for Increasing multidrug resistance among Extended-Spectrum β-Lactamase-producing Escherichia coli and Klebsiella species. Clin Infect Dis. 2005; 40: 1317–1324. 15825035 10.1086/429239

[29] Paterson DL, Bonomo R.A. Extended-spectrum *β*-lactamases: a clinical update. Clinical Microbiology Reviews. 2005; 18:657–686. 16223952 10.1128/CMR.18.4.657-686.2005 PMC1265908

[30] Kollef MH. Inadequate antimicrobial treatment: an important determinant of outcome for hospitalized patients. Clin Infect Dis. 2000; 3: S131–S138 10.1086/314079 11017862

[31] Tumbarello M, Sanguinetti M, Montuori E, Trecarichi EM, Posteraro B, Fiori B et al Predictors of mortality in patients with bloodstream infections caused by extended-spectrum beta-lactamase-producing Enterobacteriaceae: importance of inadequate initial antimicrobial treatment. Antimicrob Agents Chemother. 2007; 51:1987–1994 17387156 10.1128/AAC.01509-06 PMC1891412

[32] Tumbarello M, Sali M, Trecarichi EM, Leone F, Rossi M, Fiori B et al Bloodstream infections caused by extended-spectrum beta-lactamase producing Escherichia coli: risk factors for inadequate initial antimicrobial therapy. Antimicrob Agents Chemother. 2008; 52:3244–3252. 10.1128/AAC.00063-08 18591273 PMC2533461

[33] Hyle EP, Lipworth AD, Zaoutis TE, Nachamkin I, Bilker WB et Lautenbach E. Impact of inadequate initial antimicrobial therapy on mortality in infections due to extended-spectrum beta-lactamase producing Enterobacteriaceae: variability by site of infection. Arch Intern Med 2005; 165:1375–1380 15983286 10.1001/archinte.165.12.1375

[34] Schwaber MJ, Carmeli Y. Mortality and delay in effective therapy associated with extended-spectrum beta-lactamase production in Enterobacteriaceae bacteremia: a systematic review and meta-analysis. J Antimicrob Chemother. 2007; 60: 913–920. 17848376 10.1093/jac/dkm318

[35] Zavascki AP. Assessing risk factors for acquiring antimicrobial-resistant pathogens: a time for a comparative approach. Clin Infect Dis. 2004;39:871–872. 15472822 10.1086/423805

[36] Harris AS, Kaye SK and Carmeli Y. Reply to Zavaski. Clin Inf Dis.2004; 39:872–873

[37] Kaye KS, Harris AD, Gold H, Carmeli Y. Kaye KS, Engemann JJ et al Reference group choice and antibiotic resistance outcomes. Emerg Infect Dis. 2004; 10:1125–1128. 15207068 10.3201/eid1006.020665 PMC3323179

[38] Harris AD, Karchmer TB, Carmeli Y, Samore MH. Methodological principles of case-control studies that analyzed risk factors for antibiotic resistance: a systematic review. Clin Infect Dis. 2001; 32:1055–61. 11264034 10.1086/319600

[39] Jarlier V, Trystram D, Brun-Buisson C et al Curbing methicillin resistant Staphylococcus aureus in 38 French hospitals through a 15-year institutional control program. Arch Intern Med 2010; 170: 552–559. 10.1001/archinternmed.2010.32 20308642

[40] Haut Conseil de la Sante Publique. Recommandations relatives aux mesures à mettre en oeuvre pour prévenir l’émergence des Entérobactéries BLSE et lutter contre leur dissémination—Propositions rédigées dans l’optique de définir un programme national de prévention. 2010. Available from: http://www.hcsp.fr/explore.cgi/hcspr20100202_enterobactBLSE

[41] Surveillance and prevention of healthcare-associated infections. French national guidelines. Available from: http://www.sf2h.net/SF2H_english/SF2H_surveillance-and-prevention-guidelines-2010.pdf

[42] De Angelis G, Murthy A, Beyersmann J and Harbarth S. Estimating the impact of healthcare-associated infections on length of stay and costs. CMI. 2010;16: 1729–1735. 10.1111/j.1469-0691.2010.03332.x 20673257

[43] Beyersmann J, Kneib T, Schumacher M, Gastmeier P. Nosocomial infection, length of stay, and time-dependent bias. Infect Control Hosp Epidemiol 2009; 30:273–276. 10.1086/596020 19193018

[44] Beyersmann J, Gastmeier P, Wolkewitz M, Schumacher M. An easy mathematical proof showed that time-depended bias is inevitably leads to biased effect estimation. J Clin Epidemiol. 2008; 61: 1216–1221. 10.1016/j.jclinepi.2008.02.008 18619803

[45] Beyersmann J, Wolkewitz M, Schumacher M. The impact of time-dependent bias in proportional hazards modelling. Stat Med. 2008; 27:6439–54. 10.1002/sim.3437 18837068

[46] Barnett AG, Beyersmann J, Allignol A, Rosenthal VD, Graves N, Wolkewitz M. The time-dependent bias and its effect on extra length of stay due to nosocomial infection. Value Health 2011; 14:381–386. 10.1016/j.jval.2010.09.008 21402305

[47] Brun Buisson C, Roudot-Thoraval F, Girou E, Grenier-Sennelier C and Durand-Zaleski I. The costs of septic syndromes in the intensive care unit and influence of hospital-acquired sepsis. Intensive Care Med. 2003; 29:1464–1471 12856120 10.1007/s00134-003-1877-x

[48] Lee SY, Kotapati S, Kuti JL, Nightingale CH and Nicholau DP. Impact of Extented-Spectrum beta-Lactamase-Producing Escherichia coli and Klebsiella species on clinical outcomes and hospital costs: a matched cohort study. Infect Control Hosp Epidemiol. 2006; 27: 1226–32. 17080381 10.1086/507962

[49] World Bank data. Available from: www.data.worldbank.org/indicator.

